# Pattern of chest computerized tomography scan findings in symptomatic RT-PCR positive Covid-19 patients at the Korle Bu Teaching Hospital, Ghana

**DOI:** 10.4314/ahs.v22i2.8

**Published:** 2022-06

**Authors:** Klenam Dzefi-Tettey, Emmanuel Kobina Mesi Edzie, Philip Narteh Gorleku, Edmund Kwakye Brakohiapa, Franklin Acheampong, Abdul Raman Asemah, Henry Kusodzi, Patience Sumbawiera Saaka, Ewurama Andam Idun, Adu Tutu Amankwa

**Affiliations:** 1 Department of Radiology. Korle Bu Teaching Hospital. P.O. Box KB 77, Korle Bu, Accra. Ghana; 2 Department of Medical Imaging. School of Medical Sciences, College of Health and Allied Sciences, University of Cape Coast, P.M.B University of Cape Coast, Cape Coast. Ghana; 3 Department of Radiology, University of Ghana Medical School; 4 Korle Bu Teaching Hospital. P.O. Box KB 77, Accra, Ghana; 5 Department of Radiology, 37 Military Hospital, Accra, Ghana; 6 Department of Radiology, School of Medical Sciences, College of Health Sciences, Kwame Nkrumah. University of Science and Technology, Kumasi, Ghana

**Keywords:** Computerized Tomography Scan, COVID-19 Pneumonia, Ghana

## Abstract

**Background:**

Chest Computerized Tomography (CT) features of Corona Virus Disease 2019 (COVID-19) pneumonia are nonspecific, variable and sensitive in detecting early lung disease. Hence its usefulness in triaging in resource-limited regions.

**Objectives:**

To assess the pattern of chest CT scan findings of symptomatic COVID-19 patients confirmed by a positive RT-PCR in Ghana.

**Methods:**

This study retrospectively reviewed chest CT images of 145 symptomatic RT-PCR positive COVID-19 patients examined at the Radiology Department of the Korle Bu Teaching Hospital (KBTH) from 8th April to 30th November 2020. Chi-Squared test was used to determine associations among variables. Statistical significance was specified at p≤0.05.

**Results:**

Males represent 73(50.3%). The mean age was 54.15±18.09 years. The age range was 5 months-90 years. Consolidation 88(60.7%), ground glass opacities (GGO) 78(53.8%) and crazy paving 43(29.7%) were the most predominant features. These features were most frequent in the elderly (≥65years). Posterobasal, peripheral and multilobe disease were found bilaterally. The most common comorbidities were hypertension 72(49.7%) and diabetes mellitus 42(29.2%) which had significant association with lobar involvement above 50%.

**Conclusion:**

The most predominant Chest CT scan features of COVID-19 pneumonia were GGO, consolidation with air bronchograms, crazy paving, and bilateral multilobe lung disease in peripheral and posterior basal distribution.

## Introduction

Severe Acute Respiratory Syndrome Coronavirus 2 (SARS-CoV-2) is a highly infectious virus which causes the Corona Virus Disease 2019 (COVID-19) pneumonia^1^. Since the outbreak of the disease in Wuhan, China in late December 2019, there has been 64,455,107 confirmed cases and 1,474,415 reported deaths distributed across 220 countries and territories around the world and two international conveyances as at November 30th, 2020, 23: 16 GMT^2^.

Clinically, the disease can be asymptomatic or mild symptoms like low-level fever, fatigue, dry cough, nasal congestion, a runny nose, diarrhea and slight weakness with or without pneumonia. Severe symptoms are dyspnea and/or hypoxemia^3^.

Currently, Reverse-Transcriptase-Polymerase-Chain-Reaction (RT-PCR) is the standard test used in diagnosing COVID-19 and preferably over chest CT scan^4^, ^5^. Though findings of viral pneumonia on chest CT scan are regarded as evidence of clinical diagnosis of COVID-19 infection, the World Health Organization (WHO) does not recommend chest CT scan findings without confirmation with RT-PCR^4^. Recent studies have shown that the RT-PCR is only 30–60% sensitive although it is highly specific and during the early course of the disease it is less sensitive than chest CT scan^5^. Early triaging between patients with and without the disease is very important in the hospital setting^6^. Though chest CT scan has low specificity, it is useful in the early detection and management of COVID-19 pneumonia due to its high sensitivity^7^. In addition to being sensitive in identifying early parenchymal lung lesions, chest CT imaging is also very useful in detecting disease progression and other differential diagnosis^8^. Being one of the most readily available imaging modalities used in radiological practice in Ghana, its role in imaging of COVID-19 patients cannot be overemphasized^9^.

Ghana recorded its first two cases on the 12^th^ of March, 2020, approximately a month after the first case was recorded in Africa10. The numbers have since increased with over 51,667 confirmed cases and 323 deaths as at November 30^th^, 202011. Over this period, patients under investigation for COVID-19 have been referred to the Radiology Department of the Korle Bu Teaching Hospital (KBTH) for non-enhanced chest CT imaging studies in order to characterize their lung disease.

To date, most cases reported CT findings which include ground-glass opacities, focal consolidations, reversed halo sign, which are usually bilateral and multifocal, peripheral in distribution, and predominance in the middle, lower, and posterior lung fields^12^. In some studies, pulmonary hemorrhage, pulmonary edema, chronic obstructive pulmonary disease, bronchiolitis obliterans, and drug-induced lung disease showed similar CT features as COVID-19 pneumonia^13^, ^14^. In chronic obstructive pulmonary disease, bronchial wall thickening and lung emphysema may be visualized^15^.

CT features of COVID-19 pneumonia are nonspecific and similar to those of other lung infections, and they show variations depending on the stage of the disease onset. Therefore, it is necessary that radiologists have knowledge of the varying imaging patterns of COVID-19 and their changes during the course of the disease. We present the pattern of chest CT scan findings of symptomatic COVID-19 patients confirmed by a positive RTPCR test in Ghana.

## Methods

### Study design, setting and participants

This single-center retrospective study was conducted on an original cohort of 145 symptomatic RT-PCR positive patients who had chest CT scans at the Radiology Department of the Korle Bu Teaching Hospital (KBTH) from 8th April to 30^th^ November 2020. KBTH, located in Accra, was established in 1923 and is currently the leading referral center in Ghana and the third largest hospital in Africa^16^. The final population of 145 patients included in this study were selected consecutively with no exclusions made.

The information obtained for this study included; Age; Sex; clinical data such as symptoms and duration of onset of symptoms (in days); a positive RT-PCR test result and chest CT scan findings. Using the Ghana age structure standards, patients were grouped into one of these categories: children (≤14 years), early working age (15–24), prime working age (25–54), mature working age (55–64) and the elderly (≥65 years). The duration of onset of symptoms was categorized into four stages: stage 1 (0–4 days), stage 2 (5–9 days), stage 3 (10–14 days) and stage 4 (15–21 days).

### Image acquisition

All the non-enhanced chest CT images were acquired using a 32-slice Canon Aquilion Start (model TSX-037A, Otawara, Tochigi 324-8550, Japan) Multi Detector CT (MDCT) scanner. All scans were unenhanced with patient in supine position and images acquired during end-inspiration. The parameters used were: tube voltage 120 kV; tube current-exposure time, 300mAs; matrix 512 x 512, slice thickness and interval 1mm and 0.625mm respectively. Images were subsequently reconstructed at the workstation and transferred to the Picture Archiving and Communication System (PACS), (IBM Waston Health Global Headquarters 75 Binney St, Cambridge, MA 02141, USA).

### Image interpretation

The images were reviewed by three radiologists with more than 10 years'experience in interpreting chest CT images. Where there were disagreements between reports, consensus was reached by discussion. Reporting was done following the Radiological Society of North America (RSNA) Expert Consensus Statement on Reporting Chest CT Scan Findings of Patients related to COVID-19 pneumonia, endorsed by the Society of Thoracic Radiology, the American College of Radiology, and RSNA^17^.

Ground glass opacity (GGO), crazy-paving pattern, and pulmonary consolidation are radiological terms used and based on the standard glossary for thoracic imaging by the Fleischner Society^18^.

Lung features were described as the presence or absence of GGO, “crazy paving”, consolidation, “reversed halo” sign, cavitation, nodule and micronodule^18^–^20^. Opacity patterns were classified as predominantly ground glass, predominantly consolidation or predominantly nodular if the percentage of the particular pattern was greater than 50%^18^, ^19^. Based on distribution, these opacities were further classified as peripheral, central or mixed (peripheral and central), anterior or posterior and the lobes involved noted. Pleural changes were defined by the presence or absence of pleural thickening or pleural effusions. Bronchial changes were defined by the presence or otherwise of air bronchogram^17^, ^20^. Pneumothorax was also looked out for and documented. The degree of lobar involvement with respect to the duration of onset of symptoms was evaluated. Based on the RSNA Expert Consensus Statement on reportingnon-enhanced chest CT scan features for COVID-19 pneumonia, each non-enhanced chest CT scan report was classified as: Typical appearance, Indeterminate appearance, Atypical appearance and Negative for COVID-19 pneumonia. Typical features are multifocal rounded GGO with consolidation in a peripheral distribution with scattered areas of (“crazy paving”) and (“reversed halo”) signs but no pleural effusion or pneumothorax. Indeterminate features are multifocal non-rounded GGO with consolidation, lacking peripheral distribution, and with a diffuse, perihilar, or unilateral distribution. A typical features are multifocal (“tree-in-bud”) opacities, isolated lobar or segmental consolidation, discrete small nodules, lung cavities, or smooth interlobular septal thickening with pleural effusion (edema)^21^. Negative features are normal chest CT scan findings.

### Statistical analysis

Statistical analysis was performed using the Statistical Package for the Social Sciences (SPSS Inc. Chicago, IL, version 21.0). Continuous variables were expressed as mean and standard deviation, whereas categorical variables were expressed as counts and percentages. Tables, graphs and charts were constructed with Microsoft Excel 2010 (Microsoft Corp, Redmond, WA USA). Chi-Squared test was used to determine the associations among variables. Statistical significance for this study was specified at p≤0.05.

### Ethical considerations

The institutional review board of the KBTH approved this study with administrative approval number KBTHADM/00154/2020. Strict anonymity and confidentiality were ensured.

## Results

One hundred and forty-five (145) RT-PCR confirmed Covid-19 pneumonia patients were included in this study comprising 73(50.3%) males and 72(49.7%) females with age range of 5 months to 90 years. Majority of the patients 61(42.1%) fell within the age category of 25–54 years, followed by 49(33.8%) within the ≥ 65 years' category with just a few 3(2.1%) in the ≤ 14-year group. The average age of patients was 54.15±18.09 years. Cough (51.7%), Fever (42.8%), Dyspnea (40.7%) and myalgia (28.3%) were the most common clinical symptoms. Table 1

**Table 1:** Demographics and clinical symptoms of Patients with COVID-19 pneumonia

Item	Count (%)
**Total Patients**	145
**Sex**	
Male	73(50.3%)
Female	72(49.7%)
**Age**	
Minimum	0.42 (5 Month)
Maximum	90 years
Mean (SD)	54.15 (18.09)
**Age Group**	
≤14 years	3 (2.1%)
15–24 years	4 (2.8%)
25–54 years	61 (42.1%)
55–64 years	28 (19.3%)
≥ 65 years	49 (33.8%)
**Symptoms**	
Fever	62 (42.8%)
Cough	75 (51.7%)
Anosmia	15 (10.3%)
Sputum Production	7 (4.8%)
Dyspnea	59 (40.7%)
Myalgia	41 (28.3%)
Headache	9 (6.2%)
Gastrointestinal symptoms	4 (2.8%)
**Mean Symptoms onset in Days (SD)**	7.78 (4.554)

The elderly group (≥65years) recorded the highest frequencies in almost all the lung CT features except for micronodules. Normal or negative chest CT scan findings were most frequent 18(69.2%) in the 25–54 year group. Consolidation 88 (60.7%), GGO 78 (53.8%) and crazy paving 43 (29.7%) were the most dominant chest CT scan features across all age groups. Air bronchograms were the most common 82 (56.6%) bronchial features. Lesions were distributed mostly in bilateral, peripheral and posterior basal lobes with the elderly group (≥ 65 years) being significantly affected followed by the (25–54 year) group. The other significant associations are presented in Table 2.

**Table 2:** Age Distribution of Chest CT scan features of Patients with Covid-19 pneumonia

Item	Count (%)						P-Value

	≤14 years	15–24 years	25–54 years	55–64 years	≥ 65 years	Total	
**Patients**	3(2.1%)	4(2.8%)	61(42.1%)	28(19.3%)	49(33.8%)	145(100.0%)	-
**Lung Features**							
GGO	1(1.3%)	1(1.3%)	26(33.3%)	18(23.1%)	32(41.0%)	78 (53.8%)	0.064
Consolidation	1(1.1%)	3(3.4%)	31(35.2%)	18(20.5%)	35(39.8%)	88 (60.7%)	0.175
Crazy paving	0(0.0%)	1(2.3%)	9(20.9%)	9(20.9%)	24(55.8%)	43 (29.7%)	0.001*
Micronodules	0(0.0%)	0(0.0%)	5(33.39%)	6(40.0%)	4(26.7%)	15 (10.3%)	0.282
Reversed halo	0(0.0%)	0(0.0%)	3(15.8%)	4(21.1%)	12(63.2%)	19 (13.1%)	0.025*
Cavitation	0(0.0%)	0(0.0%)	4(44.4%)	1(11.1%)	4(44.4%)	9 (6.2%)	0.810
Septal thickening	0(0.0%)	2(5.1%)	10(25.6%)	6(15.4%)	21(53.8%)	39 (26.9%)	0.012*
No lesion(Normal features)	1(3.8%)	0(0.0%)	18(69.2%)	4(15.4%)	3(11.5%)	26 (17.9%)	0.011*
**Pleural features**							
Thickening	0(0.0%)	0(0.0%)	9(32.1%)	8(28.6%)	11(39.3%)	28 (19.3%)	0.234
Effusion	0(0.0%)	1(3.7%)	10(37.0%)	9(33.3%)	7(25.9%)	27 (18.6%)	0.276
**Bronchial** **features**							
Air bronchogram	2(2.4%)	2(2.4%)	29(35.4%)	14(17.1%)	35(42.7%)	82 (56.6%)	0.121
Bronchiectasis	0(0.0%)	0(0.0%)	4(40.0%)	2(20.0%)	4(40.0%)	10 (6.9%)	0.889
**Lesion** **distribution**							
Peripheral	1(1.1%)	2(2.2%)	29(31.2%)	20(21.5%)	41(44.1%)	93 (64.1%)	0.001*
Central	0(0.0%)	0(0.0%)	7(33.3%)	3(14.3%)	11(52.4%)	21 (14.5%)	0.265
Mixed	1(3.3%)	0(0.0%)	8(26.7%)	8(26.7%)	13(43.3%)	30 (20.7%)	0.170
Anterior	0(0.0%)	0(0.0%)	13(39.4%)	7(21.2%)	13(39.4%)	33 (22.8%)	0.386
Posterior	2(1.9%)	2(1.9%)	32(31.1%)	23(22.3%)	44(42.7%)	103 (71.0%)	<0.001*
Apical	0(0.0%)	0(0.0%)	16(35.6%)	9(20.0%)	20(44.4%)	45 (31.0%)	0.092
Basal	2(1.8%)	4(3.6%)	38(34.2%)	24(21.6%)	43(38.7%)	111 (76.6%)	0.008*
**Lesion** **predominance**							
GGO	1(1.3%)	1(1.3%)	26(33.3%)	18(23.1%)	32(41.0%)	78 (53.8%)	0.064
Consolidation	1(1.1%)	3(3.4%)	31(35.2%)	18(20.5%)	35(39.8%)	88 (60.7%)	0.175
**Pneumothorax**	0(0.0%)	0(0.0%)	1(50.0%)	1(50.0%)	0(0.0%)	2 (1.4%)	0.686
**Lymphadenopathy**							
Mediastinal	0(0.0%)	0(0.0%)	2(66.7%)	1(33.3%)	0(0.0%)	3 (2.1%)	0.562
Hilar	0(0.0%)	0(0.0%)	1(50.0%)	1(50.0%)	0(0.0%)	2 (1.4%)	0.686
**Lobar** **Involvement**							
Right Upper Lobe	0(0.0%)	0(0.0%)	20(37.0%)	8(14.8%)	26(48.1%)	54(37.2%)	0.011*
Right Middle Lobe	1(1.1%)	1(1.1%)	35(37.6%)	17(18.3%)	39(41.9%)	93(64.1%)	0.031*
Right Lower Lobe	2(1.9%)	4(3.8%)	32(30.8%)	24(23.1%)	42(40.4%)	104(71.7%)	<0.001*
Left Upper Lobe	0(0.0%)	0(0.0%)	16(32.7%)	10(20.4%)	23(46.9%)	49(33.8%)	0.026*
Left Lower Lobe	2(2.0%)	1(1.0%)	33(33.3%)	22(22.2%)	41(41.4%)	99(68.3%)	0.003*

* Statistically significant

Throughout the stages of COVID-19 pneumonia infection, bilateral multilobe lung disease with peripheral, posterior and basal distribution were the commonest findings. Table 3.

**Table 3:** Lesion distribution with the extent of lobe involvement throughout the various stages of the COVID-19 pneumonia

		Lesion Distribution (Count)						

Stages of symptom onset	Extent of Lobe Involvement	Peripheral	Central	Mixed	Anterior	Posterior	Apical	Basal
**0–4 Days**	**No Involvement**	0	0	0	0	0	0	0
	**Single Lobe**	0	0	0	0	0	0	1
	**Unilateral** **Multilobe**	0	0	1	0	1	1	0
	**Bilateral Multilobe**	15	2	5	5	18	8	18
**5–9 Days**	**No Involvement**	0	0	0	0	0	0	0
	**Single Lobe**	2	0	2	0	4	2	6
	**Unilateral** **Multilobe**	1	1	1	2	1	1	2
	**Bilateral Multilobe**	55	13	15	19	57	25	61
**10–14** **Days**	**No Involvement**	0	0	0	0	0	0	0
	**Single Lobe**	1	0	0	0	1	0	1
	**Unilateral** **Multilobe**	0	0	0	0	0	0	0
	**Bilateral Multilobe**	11	3	4	5	13	6	13
**15–21** **Days**	**No Involvement**	0	0	0	0	0	0	1
	**Single Lobe**	1	0	0	1	1	0	1
	**Unilateral** **Multilobe**	0	0	0	0	0	0	0
	**Bilateral Multilobe**	7	2	2	1	7	2	7

Note: Stages are groups based on time from initial onset of symptoms to time of chest CT scan defined by; stage 1 (0–4 days), stage 2 (5–9 days), stage 3 (10–14 days) and stage 4 (15–21 days).

37.9 % (33) of the COVID-19 pneumonia patients had more than 75% lobar involvement which occurred during stage 2 (5–9) days and had the highest number of patients (58 out of 87) with percentage lobar involvement more than 50%. Hypertension was the commonest comorbidity 72(49.7%) followed by diabetes mellitus 42(29.0 %) and these were associated with percentage lobar involvement significantly above 50%. In addition, patients with multiple comorbidities had percentage lobar involvement significantly above 50%. Table 4.

**Table 4:** Presence of comorbidity, stages of symptoms onset and percentage lobar involvement among COVID-19 pneumonia patients

Item	Count (%)						P-Value

	Percentage Lobar Involvement						

	0%	1–25 %	26–50 %	51–75 %	> 75%	Total	
**Age Group**							
≤14 years	1(33.3%)	0(0.0%)	1(33.3%)	1(33.3%)	0(0.0%)	3 (2.1%)	
15–24 years	1(25.0%)	2(50.0%)	0(0.0%)	1(25.0%)	0(0.0%)	4 (2.8%)	
25–54 years	15(24.6%)	8(13.1%)	3(4.9%)	16(26.2%)	19(31.1%)	61 (42.1%)	**0.003** *
55–64 years	4(14.3%)	0(0.0%)	8(28.6%)	9(32.1%)	7(25.0%)	28 (19.3%)	
≥ 65 years	4(8.2%)	2(4.1%)	4(8.2%)	16(32.7%)	23(46.9%)	49 (33.8%)	
**Stages of** **Symptoms onset**							
0–4 days	5(19.2%)	1(3.8%)	6(23.1%)	6(23.1%)	8(30.8%)	24 (17.9%)	
5–9 days	13(14.9%)	9(10.3%)	7(8.0%)	25(28.7%)	33(37.9%)	87 (60.0%)	0.220
10–14 days	5(23.8%)	1(4.8%)	0(0.0%)	9(42.9%)	6(28.6%)	21 (14.5%)	
15–21 days	2(18.2%)	1(9.1%)	3(27.3%)	3(27.3%)	2(18.2%)	11 (7.6%)	
**Comorbidities**							
Hypertension	6(8.3%)	1(1.4%)	6(8.3%)	28(38.9%)	31(43.1%)	72(49.7%)	**<0.001** *
Diabetes Mellitus	3(7.1%)	1(2.4%)	4(9.5%)	14(33.3%)	20(47.6%)	42(29.0%)	**0.029** *
Acute/Chronic Pulmonary Diseases	6(22.2%)	2(7.4%)	4(14.8%)	3(11.1%)	12(44.4%)	27(18.6%)	0.151
Cardiovascular Disorders	4(26.7%)	1(6.7%)	2(13.3%)	6(40.0%)	2(13.3%)	15(10.3%)	0.398
Stroke	1(20.0%)	0(0.0%)	0(0.0%)	3(60.0%)	1(20.0%)	5(3.4%)	0.466
Chronic Renal Failure	2(10.0%)	0(0.0%)	1(5.0%)	9(45.0%)	8(40.0%)	20(13.8%)	0.126
Retroviral Infection	0(0.0%)	1(25.0%)	0(0.0%)	3(75.0%)	0(0.0%)	4(2.8%)	0.093
Malignancy	1(20.0%)	2(40.0%)	1(20.0%)	1(20.0%)	0(0.0%)	5(3.4%)	0.121
Others	0(0.0%)	0(0.0%)	1(14.3%)	5(71.4%)	1(14.3%)	7(4.8%)	0.094
**Comorbidities** **Per Patient**							
0	12(36.4%)	6(18.2%)	4(12.1%)	3(9.1%)	8(24.2%)	33(22.8%)	
1	6(12.5%)	4(8.3%)	6(12.5%)	18(37.5%)	14(29.2%)	48(33.1%)	**0.009** *
2	5(10.9%)	2(4.3%)	4(8.7%)	14(30.4%)	21(45.7%)	46(31.7%)	
>2	2(11.1%)	0(0.0%)	2(11.1%)	8(44.4%)	6(33.3%)	18(12.4%)	

* Statistically significant

**Others**: 4 Deep Vein Thrombosis (DVT), 2 Systemic Lupus Erythematosis (SLE), 1 Bleeding hemorrhoids.

**Figure 1:**
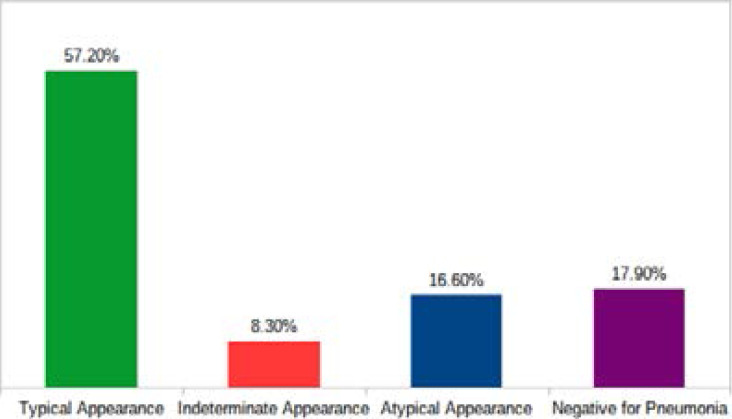
Classification of the chest CT scan features of COVID-19 pneumonia (Based on the RSNA Expert Consensus Statement on reporting non-enhanced chest CT scan features for PUI COVID-19 pneumonia).

In assessing the overall chest CT scan features for the COVID-19 pneumonia patients based on the RSNA Expert Consensus Statement on reporting non-enhanced chest CT scan features for PUI for COVID-19 pneumonia, we found that majority of the patients 80(55.20%) out of 145 had typical features for Covid-19 pneumonia Figure 1.

## Discussion

Chest CT imaging results of RT-PCR positive patients with COVID-19 pneumonia have been reported. The most common symptoms were cough, fever, and dyspnea which took an average of 7.78 (±4.554) days to manifest. These symptoms are arguably the common presentation among patients with COVID-19 pneumonia worldwide^8^, ^22^–^24^.

The CT imaging features from this study were GGO, consolidation, crazy paving pattern, air bronchogram signs, and intralobular septal thickening. These lung features were predominant in the elderly population (Table 2). Our result is consistent with a study in Rome, Italy, conducted by Damiano et al on 158 patients comprising 83 males and 75 females. The cohort in their study showed a higher frequency of pulmonary consolidations (60.7% against 72.0%) and GGO (53.8% against 100%)25. Zhu et al also reported only 47% of GGO in patients with COVID-19 from a population of 32 symptomatic patients26. Recently, Chung et al analyzed a small population of 21 patients and found a very low frequency of crazy paving pattern compared with our results (4 patients against 43 patients)^27^. There were no signs of reticulation, calcification and tree-in-bud appearance in any of our patients and only a few of the patients had bronchiectasis. The absence of these imaging features have also been demonstrated by other studies^28^–^30^. In one study, they found that calcification, bronchiectasis, cavitation, reticulation, reversed halo-sign, tree-in-bud appearance, and nodules were absent iall patients except one who had pleural effusion^29^. The authors argued that these lung features normally develop and it may not be possible to arrive at a conclusion through investigation of early imaging and hence was considered as a late finding. They further added that invasive fungal infections like aspergillosis, vasculitis and even tumor metastasis can have reversed halo sign^29^.

Our study also showed that, over all, the lobes more frequently involved are the lower lobes. Song et al. in their study also found lower lobe involvement in 90% of COVID-19 pneumonia patients which is consistent with what we found, whilst Shi et al. also had similar finding with peripheral and lower lobe involvement^31^, ^32^. In addition, a study in China among 102 patients with COVID-19 revealed that the most involved sites were the posterior basal segments of the right and lower lobes^33^. They did not however report the difference of lobe involvement and distribution with respect to age. Our study revealed that certain lobes were more frequently involved in older patients (Table 2). This might give clues to radiologists to be very observant when reviewing the images of these populations; however, more studies are needed. Most of our findings were bilateral, peripheral, posterior and basal distribution of lung lesions on imaging. Other studies have reported this finding^31^, ^34^. However, in contrast to our study, Chung et al reported only 33% of peripheral distribution^27^.

In evaluating the presence of the predominant CT abnormalities with time (between onset of symptoms and chest CT imaging) in confirmed cases of COVID-19, we realized that abnormalities were more manifested in the group with onset of symptoms between 5 – 9 days and least in those between 10 – 14 days. Further, we found that bilateral multi-lobar disease was more common among those we evaluated between 5 – 9 days after onset of symptoms. Multi-lobar involvement after being at its peak in the 5 - 9 days' onset of symptoms group, appeared to decline in patients with onset of symptoms of 10 days and beyond (Table 3). In a study by Pan et al. to investigate lung changes of COVID-19 pneumonia with time, it was highlighted that the degree of lung involvement increased with time reaching a peak between 9 – 13 days after onset of symptoms and steadily declined thereafter35. However, the differences in the time period within which the maximum CT abnormalities were found, may suggest several factors among which could be a probable faster progression of lung disease in our study population compared to the study earlier cited, an area which may need further investigation.

Comorbidities associated with COVID-19 have been reported for some diseases such as hypertension, renal diseases, cardiovascular diseases, chronic respiratory diseases, chronic kidney diseases, malignancies, and diabetes mellitus^36^. We found hypertension and diabetes mellitus to be the most common comorbidities with a percentage lobar involvement above 50%. The number of these comorbidities were significantly associated with the severity of lobar involvement (Table 4). Huang and Wang et al also found hypertension to be the common type of comorbidity associated with COVID-19 followed by diabetes mellitus^1^, ^12^. This was also corroborated by a recent study in Ghana^37^. Understanding the relationship between comorbidities and COVID-19 will not only improve clinical decisions but will enable understanding of how to manage complications for the population at risk. As part of our study, we classified each patient's chest CT imaging findings as Typical, Atypical, Indeterminate and Negative for COVID-19 pneumonia using the RSNA consensus statement on structured reporting of CT features of PUI for COVID-19 pneumonia. Standardized reporting provides guidance to radiologists as well as increased clarity^21^. We found that, most patients showed typical features of COVID-19 pneumonia on chest CT scan. Standardized radiology reporting in association with clinical evaluation will help future care algorithms to predict which patients will have RT-PCR test should testing become a challenge. Initial RT-PCR test may also be negative, and typical imaging features should encourage repeat testing, isolation and treatment^38^.

The relatively small sample size and the inability to do follow up chest CT scans for disease progression or resolution are the limitations of the study.

## Conclusion

Chest CT scan features of COVID-19 pneumonia were bilateral, ground-glass opacities with air bronchograms, and predominantly in bilateral, peripheral and posterior basal distribution. The clinical presentation, course, and outcome of COVID-19 pneumonia are heterogeneous, and this is also related to the degree of pulmonary involvement and comorbidities. Chest CT scan features commonly seen in COVID-19 pneumonia are GGO, consolidation with air bronchograms, lower lobe involvement, bilateral and posterior predilection and this knowledge may help radiologists in making diagnosis. As COVID-19 infection continues to rise, radiologists and radiographers will encounter patients with varying presentations of this infection, therefore the need to be familiar with the typical clinical and chest CT imaging features and its changes during the course of the disease in order to interpret images and identify cases during imaging which will enable optimal triaging, isolation, contact tracing and management.

## Figures and Tables

**Figure 2:**
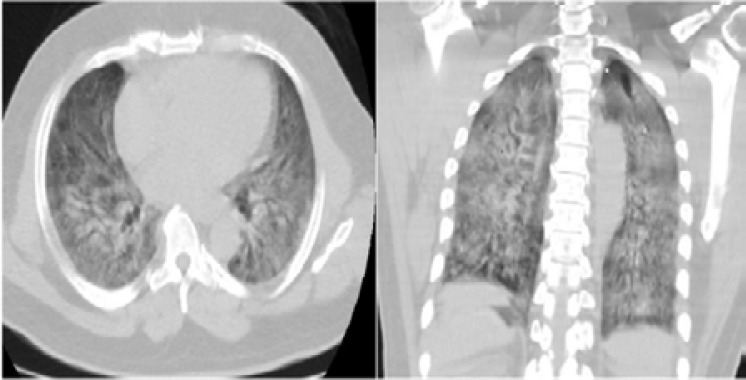
Axial and coronal reformatted unenhanced chest CT scan of a 63-year -old man with positive RT-PCR test and 7-days history of fever, cough and dyspnea showing consolidation with air bronchograms in bilateral posterior basal distribution; typical features of COVID-19 pneumonia.
